# Hantavirus pulmonary syndrome in a Chilean patient with recent travel in Bolivia.

**DOI:** 10.3201/eid0401.980112

**Published:** 1998

**Authors:** R Espinoza, P Vial, L M Noriega, A Johnson, S T Nichol, P E Rollin, R Wells, S Zaki, E Reynolds, T G Ksiazek

**Affiliations:** Clinica Alemana de Santiago, Chile.

## Abstract

A case of hantavirus pulmonary syndrome (HPS) was serologically confirmed in a critically ill patient in Santiago, Chile. The patient's clinical course had many similarities to that of other HPS patients in North and South America but was complicated by acute severe renal failure. The patient's history included self-reported urban and probable rural rodent exposure during travel in Bolivia. Comparison of a viral sequence from an acute-phase serum sample with other known hantaviruses showed that the hantavirus nucleic acid sequence from the patient was very similar to a virus recently isolated from rodents associated with HPS cases in Paraguay.


### Hantavirus Pulmonary Syndrome in a Chilean Patient with Recent Travel in Bolivia

A case of hantavirus pulmonary syndrome (HPS) was serologically confirmed in a critically ill patient in Santiago, Chile. The patient's clinical course had many similarities to that of other HPS patients in North and South America but was complicated by acute severe renal failure. The patient's history included self-reported urban and probable rural rodent exposure during travel in Bolivia. Comparison of a viral sequence from an acute-phase serum sample with other known hantaviruses showed that the hantavirus nucleic acid sequence from the patient was very similar to a virus recently isolated from rodents associated with HPS cases in Paraguay.

Since its discovery in 1993 (1) and its association with Sin Nombre virus in North America (2), hantavirus pulmonary syndrome (HPS) has been identified in several countries in South America and is associated with Juquitiba virus in Brazil (3), Andes virus in Argentina and Chile (4-6), and Laguna Negra virus in Paraguay (7,8). Hantaviruses are rodent-borne, and each is associated with a specific primary rodent reservoir. Sigmodontine rodents are the vectors of hantaviruses associated with HPS (3).

### Case Report

A 20-year-old male resident of Santiago, Chile, who had no prior history of medical problems, became ill after he backpacked (from February 4 to March 9, 1997) as a tourist in Bolivia. His travel itinerary included Barrio, Oruro, La Paz, Cochabamba, Villa Tunari, Santa Cruz, Vallegrande, Higueras, and Sucre. The tourist and a fellow traveler stayed in hotel in Santa Cruz where they saw black rats running through the bathroom they used. While in Higueras, they stayed in a rustic adobe house with no floor and joined local villagers in agricultural jobs, primarily harvesting hay. A diagnosis of HPS was considered.

On March 26, 3 weeks after returning to Chile, the young man became ill with fever and cough. On March 28, he was admitted to the emergency room of a private hospital in Santiago with high fever (39°C), cough, and chest pain. Chest X-rays showed interstitial infiltrates in the left lower lobe. Laboratory results were as follows: hemoglobin 15.3 gm/dl, white blood cells 5,900, platelets 130,000, erythrocyte sedimentation rate 6, and a C-reactive protein 2.8 mg/dl. He was sent home on clarithromycin 250 mg, twice a day. Three days later (March 31), he returned to the emergency room with persistent high fever, myalgia, and shortness of breath; distal cyanosis was noted. Vital signs were as follows: blood pressure 115/80, pulse 120, temperature 38.5°C, and respiratory rate 32. Physical examination found few petechiae on the forearms, diffuse bilateral rales, regular cardiac rhythm, no murmurs, and no hepatomegaly or splenomegaly. Chest X-rays showed diffuse bilateral alveolar infiltrates. Oxygen saturation was 90%. Laboratory values included white blood cells 11,700 with a left shift, platelets 150,000, hemoglobin 19.4, erythrocyte sedimentation rate 2, C-reactive protein 8.18, INR 1.6, activated partial thromboplastin 43s, blood urea nitrogen 38.9 mg/dl, and liver function tests normal limits.

The patient was transferred to the intensive care unit with a diagnosis of bilateral pneumonia of unknown etiology and secondary respiratory failure. In 3 to 4 hours, acute respiratory distress developed; arterial blood gases (on 50% oxygen) were $PaO_2$ 61, $PaCO_2$ 22.7, pH 7.49, and $HCO_3$ 17.1. The patient was connected to a ventilator and was started on imipenen, erythromycin, amantadine, dopamine, dobutamine, fluids, and nitric oxide. Two 500-mg doses of methylprednisolone were administered, and a Swan-Ganz catheter was installed. The pulse wedge pressure was 7, and the cardiac output 6.1. During the first 24 hours, acute renal failure developed, and hemodialysis was started.

Hypotension was quite refractory to vasoactive drugs (mean BP 30 to 40), and critical hypoxemia ($O_2$ saturation 70% to 80%) and hypotension persisted for 48 hours. Echocardiography showed a mild pericardial effusion. Blood cultures were negative for bacteria, as well as for influenza, adenovirus, respiratory syncytial virus, and parainfluenza virus. Serologic results for *Mycoplasma*, *Legionella*, and HIV were also negative. Blood gases started to improve on day 3, and the patient's clinical condition also slowly improved. On day 10, hemolytic anemia (Coombs negative) developed, and a bone marrow aspirate showed hemophagocytosis. On day 11, an episode of bleeding from the respiratory tract occurred. However, the patient's ventilatory function continued to improve. On day 15 after admission, his chest X-ray was clearing, but he was still on a mechanical ventilator ($O_2$ saturation 98% on 40% $O_2$). However, he was anuric (BUN 100). On day 17, he had a second episode of bronchial bleeding, and bronchoscopy showed only a 4-mm tracheal ulcer. Platelets were 72,000, and hematocrit fell to 24%. Hantavirus serology was reported positive [Centers for Disease Control and Prevention (CDC), April 18], and intravenous ribavirin was started on April 22. A central venous catheter-related infection with *Pseudomonas* and *Staphylococcus aureus* was documented. The patient's condition deteriorated progressively, with further bronchial bleeding and markedly unstable ventilatory function, and required constant administration of vasoactive drugs; he died of massive pulmonary hemorrhage and shock on April 28. A postmortem lung sample was taken with a needle.

The patient's serum, collected on April 1, was tested for immunoglobulin G (IgG) and IgM antibodies to Sin Nombre virus at CDC. Both IgG and IgM antibodies were found, which suggested recent infection with a hantavirus associated with Sigmodontine rodents. Subsequent testing of sera collected on April 21 and April 25 confirmed the initial findings. Hematoxylin and eosin stained sections of the lung sample showed diffuse alveolar damage with extensive hyaline membrane formation, proliferation of type II pneumocytes, and fibroblastic and edematous thickening of alveolar walls. Abundant fibrin, necrotic debris, and acute inflammatory cellular infiltrates were also observed in the alveolar spaces. Rare endothelial cells and macrophages were hantavirus-antigen positive by previously described immunohistochemical procedures (9). The destructive changes seen by histopathology and a small amount of antigen found in the patient's tissues are compatible with the long course of the patient's illness (9).

Viral RNA was extracted from the earliest serum sample, and reverse transcriptase-polymerase chain reaction amplification with primers designed specifically for hantaviruses associated with Sigmodontine rodents (8) yielded polymerase chain reaction fragments for the S and M segments. These cDNA fragments were sequenced and compared with those of other American hantaviruses. These comparisons show that the virus is closely related to other South American hantaviruses, and most closely related to Laguna Negra virus detected in patients and *Calomys laucha* rodents (vesper mice) in the Chaco region of Paraguay (7,8). The nucleotide sequence identity was 84% for the G1 protein encoding fragment and 87% for the N protein encoding fragment. However, the deduced amino acid sequences for both fragments were identical to Laguna Negra virus. This shows that the virus associated with this HPS case is a Laguna Negra virus variant and suggests that the virus is probably associated with the same or a very closely related species of rodent host.

*C. laucha*, the apparent primary rodent reservoir for Laguna Negra virus, is common in savanna and grassland areas as far north as southern Brazil and Bolivia, throughout much of Paraguay and Uruguay, and in Argentina as far south as Rio Negro province, but not in Chile (10). A number of the lowland rural locations that the patient visited in Bolivia are within the range of *C. laucha*.

The clinical course and travel history of the patient and the laboratory serology and molecular characterization of the viral RNA are compatible with infection in Bolivia with a Laguna Negra virus variant. This report and evolving information concerning hantaviruses associated with clinical HPS in Argentina and Paraguay strongly suggest that a diagnosis of HPS should be considered in patients with febrile respiratory distress syndrome throughout Latin America.

**Ricardo Espinoza,* Pablo Vial,*† Luis M. Noriega,*† Angela Johnson,‡ Stuart T. Nichol,‡ Pierre E. Rollin,‡ Rachel Wells,‡ Sherif Zaki,‡ Enrique Reynolds,* and Thomas G. Ksiazek‡**

*Clínica Alemana de Santiago, Santiago, Chile; †Escuela de Medicina, Universidad Católica de Chile, Santiago, Chile; ‡Centers for Disease Control and Prevention, Atlanta, Georgia, USA.

### References

1. Duchin JS, Koster F, Peters CJ, Simpson G, Tempest B, Zaki S, et al. Hantavirus pulmonary syndrome: a clinical description of 17 patients with a newly recognized disease. N Engl J Med 1994;330:949-55.
2. Nichol ST, Spiropoulou CF, Morzunov S, Rollin PE, Ksiazek TG, Feldmann H, et al. Genetic identification of a hantavirus associated with an outbreak of acute respiratory illness. Science 1993;262:914-7.
3. Nichol ST, Ksiazek TG, Rollin PE, Peters CJ. Hantavirus pulmonary syndrome and newly described hantaviruses in the United States. In: Elliott RM, editor. The Bunyaviridae. New York: Plenum Press, 1996:269-80.
4. Lopez N, Padula P, Rossi C, Lazaro ME, Franze-Fernandez MT. Genetic identification of a new hantavirus causing severe pulmonary syndrome in Argentina. Virology 1996;220:223-6.
5. Centers for Disease Control and Prevention. Hantavirus pulmonary syndrome in Chile—1997. MMWR Morb Mortal Wkly Rep 1997;46:949-51.
6. Lopez N, Padula P, Rossi C, Miguel S, Edelstein A, Ramirez E, et al. Genetic characterization and phylogeny of Andes virus and variants from Argentina and Chile. Virus Research 1997;50:77-84.
7. Williams RJ, Bryan RT, Mills JN, Palma RE, Vera I, de Velasquez F, et al. An outbreak of hantavirus pulmonary syndrome in Western Paraguay. Am J Trop Med Hyg 1997;57:274-82.
8. Johnson AM, Bowen MD, Ksiazek TG, Williams RJ, Bryan RT, Mills JN, et al. Laguna Negra virus associated with HPS in western Paraguay and Bolivia. Virology. In press 1997.
9. Zaki S, Greer PW, Coffield LM, Goldsmith CS, Nolte KB, Foucar K, et al. Hantavirus pulmonary syndrome: pathogenesis of an emerging infectious disease. American Journal of Pathology 1995;146:552-79.
10. Redford KH, Eisenberg JF. Mammals of the neotropics, the southern cone. Chicago: The University of Chicago Press, 1992:279-80.
